# Increased importin 13 activity is associated with the pathogenesis of pterygium

**Published:** 2013-03-20

**Authors:** Ke Xu, Tao Tao, Jing Jie, Xiaodong Lu, Xuezhi Li, Muhammad Aamer Mehmood, Hui He, Zhen Liu, Xinye Xiao, Jie Yang, Jian-xing Ma, Wei Li, Yueping Zhou, Zuguo Liu

**Affiliations:** 1 Eye Institute of Xiamen University, Fujian Provincial Key Laboratory of Ophthalmology and Visual Science; School of Life Science of Xiamen Universtity, Key Laboratory for Cell Biology and Tumor Cell Engineering of The Ministry of the Education of China, Xiamen, China; 2 Eye Institute and affiliated Xiamen Eye Center of Xiamen University, Fujian Provincial Key Laboratory of Ophthalmology and Visual Science, Xiamen, Fujian, China; 3 School of Life Science of Xiamen Universtity, Key Laboratory for Cell Biology and Tumor Cell Engineering of The Ministry of the Education of China, Xiamen, China; 4 Department of Bioinformatics and Biotechnology, Government College University Allama Iqbal road Faisalabad, Pakistan; 5 Department of Physiology, The University of Oklahoma Health Sciences Center, Oklahoma City, OK

## Abstract

**Purpose:**

We previously reported that importin 13 (IPO13), a member of the importin-β family of nuclear import proteins, regulates nuclear import of the glucocorticoid receptor in airway epithelial cells, IPO13 serves as a potential marker for corneal epithelial progenitor cells, and IPO13 is associated with corneal cell proliferation. Here we investigated the role of IPO13 in the pathogenesis of pterygium and the underlying mechanism including interaction with other cell proliferation–related factors: keratin 17 (K17), a lesional protein and a member of the type I keratins, and c-Jun, a protein of the activator protein-1 complex.

**Methods:**

Tissue samples were collected from primary pterygia, recurrent pterygia, and normal conjunctiva to perform the following experiments: immunohistochemical measurement of IPO13 and K17. Pterygium epithelial cells (PECs) were cultured in keratinocyte serum-free defined medium to examine the expression of IPO13 and K17. Lentivirus-mediated silencing and overexpression IPO13 testing was conducted, and K17 alternation was evaluated with western blot and immunostaining. In addition, the translocation of c-Jun (a K17 regulator) was further examined after IPO13 was silenced.

**Results:**

IPO13 activity was significantly increased in the basal layer of the epithelium of the pterygium. In cultured PECs, overexpression or knockdown of the *IPO13* gene increased or decreased PEC proliferation, respectively. IPO13 was colocalized with K17 in the epithelium of the pterygium, and overexpression or knockdown of the *IPO13* gene induced upregulation or downregulation of K17 expression in PECs, respectively. In addition, silencing of the *IPO13* gene blocked nuclear translocation of c-Jun.

**Conclusions:**

We provided novel evidence that IPO13 may contribute to the pathogenesis of pterygium via modulation of K17 and c-Jun.

## Introduction

Pterygium is one of the most common ocular surface diseases. Millions of people have pterygia worldwide, particularly in specific regions, for example, Australia and Southeast Asia. A pterygium is a fibrovascular neoformation characterized as triangular wing-shaped overgrowth of abnormal conjunctiva onto the cornea and is composed of epithelium and highly vascular, sub-epithelial, loose connective tissue [1]. In severe cases, a pterygium can grow into the central cornea, induce irregular corneal astigmatism, and even result in vision loss [2]. Currently, the main therapeutic method is surgical removal with a high rate of recurrence [3]. The pathogenesis and mechanism of the pterygium remain largely unknown. Pterygia share many similar traits with tumor and neoplasia such as proliferation, invasion, and recurrence after resection [4]. Recently, a pterygium was considered a type of hyperproliferation of the conjunctival epithelium [5-7].

Nucleocytoplasmic transport is an essential phenomenon in eukaryotic cells controlled by the family of proteins called importins and exportins. Importin 13 (IPO13), a member of the importin-β superfamily, was originally identified in the fetal rat lung and differentially expressed in cells of epithelial and mesenchymal origin. Recently, IPO13 was reported to play a role in human physiologic and pathologic function. Several investigators found that IPO13 is responsible for embryonic development in the lungs, brain, and heart [1,2], suggesting that the nucleocytoplasmic transport biofunction involves corresponding physiologic events. With several of IPO13’s specific cargoes such as pax6 and ubiquitin-conjugating enzyme [1,3] identified, elucidating the mechanism underlying the physiologic and pathologic functions and interactions with other factors has become important. Variations in the *IPO13* gene or alteration in IPO activity is associated with various diseases, such as endometrial polyps [4] and endometrial carcinoma [5]. We also recently reported that IPO13 regulates nuclear import of the glucocorticoid receptor in airway epithelial cells [6], IPO13 serves as a potential marker for corneal epithelial progenitor cells, and IPO13 is associated with corneal cell proliferation [7]. The role of IPO13 in ocular epithelial diseases such as pterygium has not been previously reported.

Keratin, a family of fibrous structural proteins, is the important structure component of certain organisms such as skin, hair, and nail. Abnormal performance of keratin such as overexpression or absence is almost contingent on the disorder of the organism itself. Especially, some keratin proteins have been documented in the study of the pathological process of pterygium lately, and were predominantly defined in the epithelium layer [8,9]. As a type I keratin, keratin 17 (K17) is associated with several skin diseases [10,11] and is present in various carcinomas [12]. Overexpression of K17 is correlated with a poor prognosis in breast [13,14] and pancreatic cancers [15]. Recently, multiple studies demonstrated that K17 promoted cell proliferation, and K17, as a pathogenic effector, was involved in disease occurring or deteriorating due to the protein’s proliferative feature [16-18]. K17 is also correlated with robust inflammation and epithelium proliferation. It has not been documented that K17 is associated with the pathogenesis of pterygium. In the present study, we for the first time investigated the role of IPO13 in the pathogenesis of pterygium and the underlying mechanism including interaction with other cell proliferation–related factors, such as K17, a type I keratin [19], and c-Jun, a protein of the activator protein-1 (AP-1) complex [20].

## Methods

### Patients and tissues

Thirty-two patients (20 with primary pterygia and 12 with recurrent pterygia) irrespective of sex (10 cases of men and 22 cases of women) and age (43–65 years old) were recruited. The method of specimen collection was performed as previously described [21]. The pterygium tissue from a patient was excised from the head (a pterygium invading the cornea). The patients were not found any severe ocular complications, e.g. corneal ulcer etc. when recruited. The patients underwent pterygium surgery at Xiamen Eye Center. All investigations were conducted in accordance with the tenets of the Declaration of Helsinki and were approved by the Ethics Committee of Xiamen Eye Center (affiliated hospital of Xiamen University). Written informed consent was acquired from all participating patients. Normal human conjunctival tissues were obtained from the Xiamen eye bank, and stored in supplemental hormonal epithelial medium at 4 °C for less than 2 h. All tissue samples were immediately used for the experiment.

### Immunostaining

Antibodies: The monoclonal antibody for K17 was purchased from DAKO (Glostrup, Denmark). The polyclonal antibody for IPO13 was generated by our group (Dr. Tao’s lab) [2]. The antibodies for c-Jun were purchased from Abcam (Cambridge, MA). Alexa Fluor 488-conjugated and Alexa Fluor 546-conjugated immunoglobulin G (IgG; antirabbit, antimouse) were purchased from Invitrogen (Carlsbad, CA). The β-actin antibody was purchased from Bio-Rad (Hercules, CA).

Conjunctival and pterygium tissue samples were sectioned with a Microm HM550 cryostat (Microm, Walldorf, Germany) at 6 μm thickness. Frozen sections were fixed in 4% paraformaldehyde in phosphate buffered saline (PBS; 137 mM NaCl; 2.7 mM KCl; 10 mM Na_2_HPO_4_; 2 mM KH_2_PO_4_) at room temperature for 15 min. For immunohistochemical staining, the endogenous peroxidase activity was inhibited with 0.3% hydrogen peroxide in PBS for 10 min. Sections were incubated with 0.2% Triton X-100 in PBS for 15 min and then incubated with 2% bovine serum albumin to block the nonspecific sites. Primary antibodies K17 (1:50) and IPO13 (1:400) were applied and incubated at 4 °C for 16 h. After three washes with PBS for 15 min, the sections were incubated with biotinylated antimouse mouse IgG (1:500) or antirabbit rabbit IgG (1:500) using Vectastain Elite ABC kits (Shanghai, China) following the manufacturer’s instruction. The reaction products were then developed with diaminobenzidine, the peroxidase substrate for 2 min, and counterstained with hematoxylin. The sections were mounted with mounting medium and examined with a Nikon Eclipse 50i microscope (Tokyo, Japan).

For immunofluorescent staining, the frozen sections, the corneal and limbal epithelial clonal culture, as well as pterygium epithelial cells (PECs) were fixed with 4% paraformaldehyde at room temperature and permeated with 0.2% Triton X-100 in PBS for 15 min. After three rinses with PBS (5 min each) and preincubation with 2% bovine serum albumin in PBS for 30 min, the IPO13 (1:400), K17 (1:50), and c-Jun (1:100) antibodies were then applied and incubated at 4 °C for 16 h. After three washes with PBS (5 min each), Alexa Fluor 488-conjugated and Alexa Fluor 546-conjugated secondary antibodies (1:500) were applied at 37 °C for 1 h in a dark incubation chamber. After washes, the sections were mounted with an antifade solution with 4’,6-diamino-2-phenylindole dihydrochloride and photographed with a Nikon Eclipse TE-2000U epifluorescent microscope (Tokyo, Japan) using a DMX 1200 digital camera (Garden City, NY).

### Culture of pterygium epithelium cells

Fresh pterygial specimens were cut into small pieces (1–2 mm in diameter) under a stereomicroscope, washed in keratinocyte serum-free defined medium (KSFM; Gibco-BRL, Grand Island, NY), and placed in a culture dish. The culture was placed in a CO_2_-regulated incubator with 5% CO_2_. Medium was added to cover the explants. The culture medium was replaced three times a week after the appearance of an outgrowth of cells. The cell type was further confirmed with immunostaining using pan cytokeratin antibodies (data not shown).

### Subcellular fractionation

Subcellular fractionation was performed using the NE-PER nuclear and cytoplasmic extraction kit (Pierce, Rockford, IL) following the manufacturer’s instruction except that all extraction reagents were supplemented with a proteasome inhibitor cocktail (Roche, Indianapolis, IN) and ubiquitin aldehyde (Sigma, St. Louis, MO) to prevent degradation and deubiquitylation.

### Gene transfection

The DNA oligos encoding human IPO13-short hairpin RNA (shRNA; Table 1) were synthesized and cloned into the pLV-H1-EF1α-puro (Biosettia Inc., San Diego, CA) vector to generate the lentiviral RNAi vectors (small interfering RNA [siRNA]-IPO13; BLOCK-iTRNAi designer Invitrogen). Lentivirus that expressed shRNA targeted against irrelevant gene luciferase (siRNA-CTR) was used as a negative control. The pcDNA3.1-LGL2-HA was generated by our group (Dr. Tao’s lab) [2]. IPO13 was amplified with PCR from the plasmid using specific primers and cloned into the BamHI and XbalI sites of pLV-EF1α-cs2-N-Flag (Biosettia).

**Table 1:** Primers used in the present study.

Primer	Nucleotide sequence (5’-3’)
IPO13-ShRNA	AAAAGTGGAGGAGATCCTTAAGATTGGATCCAATCTTAAGGATCTCCTCCAC
K17-Forward	TGGAGCAGCAGAACCAGGAATACA
K17-Reverse	TCTTCCACAATGGTACGCACCTGA
IPO13- Forward	TCAAGGCCTCCTGTGGCTTCTTTA
IPO13- Reverse	AGCACTGCTATGAGCAGCATACGA
GAPDH- Forward	AGCCTCAAGATCATCAGCAATGCC
GAPDH- Reverse	TGTGGTCATGAGTCCTTCCACGAT
pLV-flag-IPO13-Forward	GCTCTAGAATGGAGCGGCGGGAGGAG
pLV-flag-IPO13-Forward	CGCGGATCCTCAGTAGTCAGCTGTGTAGT

The lentiviruses were produced by cotransfecting 293FT cells (5×10^6^ per plate) with lentiviral vector and packaging plasmids (Lipofectamine 2000, Invitrogen). Supernatants were collected after 48 h of transfection and filtered, and viral titers were determined with fluorescence-activated cell sorter. Target cells were infected with lentivirus in the presence of 8 μg ml^−1^ polybrene (Sigma-Aldrich) [22,23]. After 48 h of incubation at 37 °C, the cells were harvested and subjected to western blot and quantitative real-time PCR, respectively.

### 3-(4,5-dimethylthiazol-2-yl)-2,5-diphenyltetrazolium bromide assay

PECs were cultured in KSFM. For cell viability assay, PECs were plated at a density of 5×10^3^ cells per well in 96-well culture plates. When the PECs were cultured up to 50% confluency, the medium was removed and transfected with siRNA-CTR, siRNA-IPO13, pLV-flag, and pLV-flag-IPO13. 3-(4,5-dimethylthiazol-2-yl)-2,5-diphenyltetrazolium bromide (MTT) assay was performed after 48 h. Briefly, 100 μl of 1 mg/ml MTT constituted in culture media was added into each well, and followed by incubation for 4 h at 37 °C in the dark. After incubation, the MTT solution was removed, and then the stained cells were washed twice in PBS followed by air-drying. The MTT-formazan products were extracted with 100 μl of dimethyl sulfoxide in the dark at room temperature. The absorbance was measured spectrophotometrically at 570 nm using a Bio TekELX800 microplate reader (Biotek Instruments, Winooski, VT).

### Quantitative real-time polymerase chain reaction

Quantitative real-time PCR was performed in a 20 μl reaction mixture containing 1 μl template DNA, 0.15 μM of each primer, and 10 μl of AmpliTaq SuperMix (Applied Biosystems) with ROX and SYBR Green I. The reaction was performed using a 7500 Real-time System (Applied Biosystems) in the following procedure: 2 min incubation at 95 °C, 10 min incubation at 55 °C, followed by 40 cycles consisting of 15 s at 95 °C, 1 min at 60 °C followed by the plate read. The cycle threshold was set using the 7500 system software, version 1.3 (Applied Biosystems). The primers (Table 1) targeting the K17, IPO13, and glyceraldehyde 3-phosphate dehydrogenase genes were designed by Primerequest.

### Western blotting

Samples were electrophoresed on 10% sodium dodecyl sulfate–polyacrylamide gels (29:1) and transferred to polyvinylidene ﬂuoride membrane (no. 162–0177; Bio-Rad) using transfer buffer (39 mM glycine, 48 mM Tris, 0.037% sodium dodecyl sulfate, 10% methanol) for 1 h. Membranes were then blocked in TBS-T buffer (20 mM Tris-HCl, pH 7.6, 137 mM NaCl, 0.1% Tween-20) containing 5% skim milk at room temperature for 1 h. After blocking, primary antibodies for IPO13 (1:1500), K17 (1:250), HDAC1 (1:500), β-tubulin (1:5000), and c-Jun (1:400) were applied in the same blocking solution at room temperature for 2 h. Extensive washes with TBS-T were performed between incubations to remove non-specific binding. The protein bands were visualized using enhanced chemiluminescence (NEN Life Science Products Inc., Boston, MA).

### Statistical analysis

Statistical analysis of western blotting and quantitative real-time PCR in the pterygium and normal conjunctiva was performed with one-way analysis of variance (ANOVA). The post hoc analysis Tukey’s test was used to compare the differences between the groups. The MTT data were analyzed with the Student *t* test. p<0.05 was considered statistically significant between the groups.

## Results

### Importin 13 expression was increased in the pterygium epithelium

**Figure 1:**
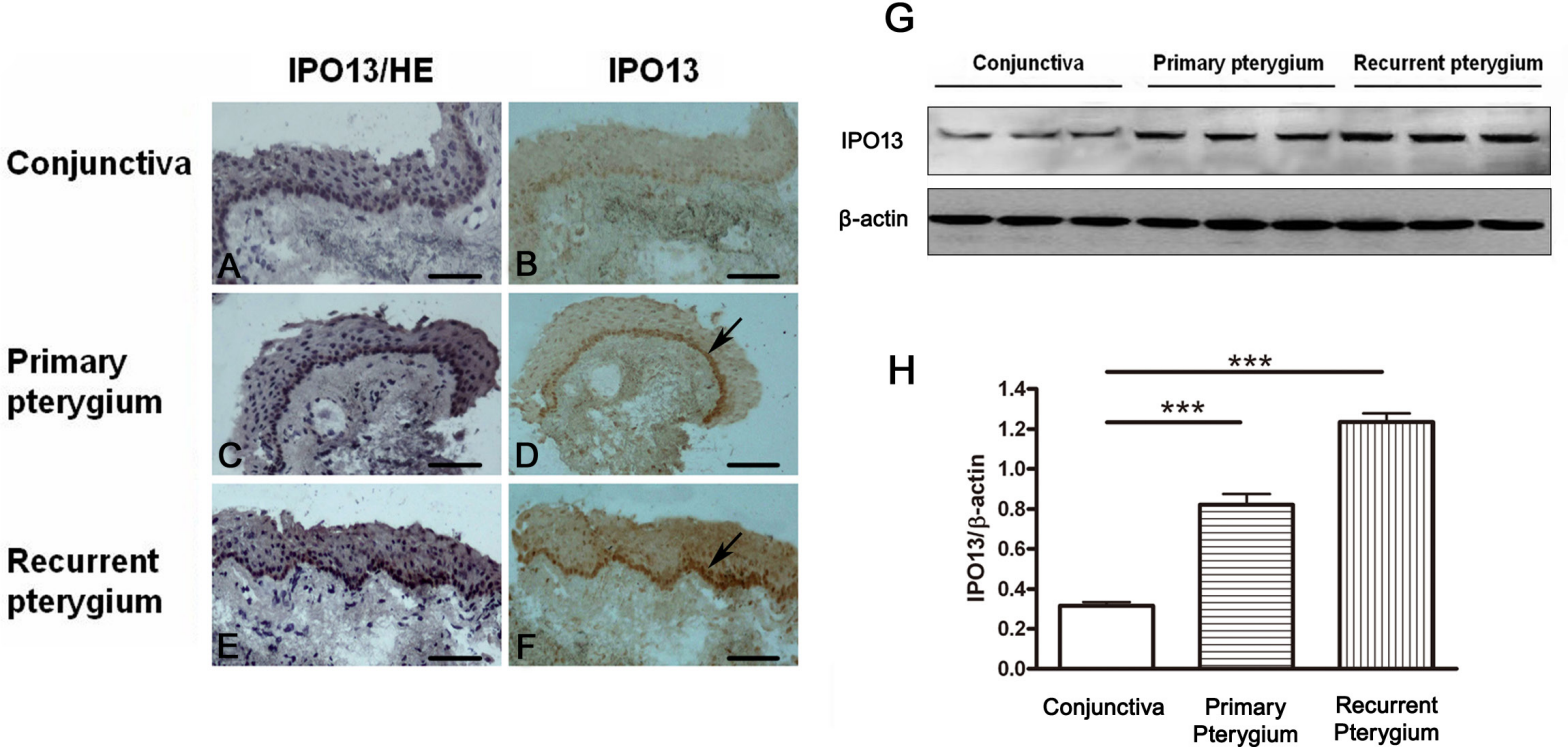
Importin 13 (IPO13) expression was increased in the epithelium of the pterygium. **A**-**F** demonstrates representative images of immunohistochemical staining of IPO13 in the normal conjunctiva (**A**, **B**), primary pterygium (**C**, **D**) and recurrent pterygium (**E**, **F); A**, **C**, **E** demonstrate representative images of H&E staining indicating the nuclei in normal conjunctiva and pterygium respectively. It was shown that the expression of IPO13 in the basal layer of epithelium cells was apparently increased (**D**, **F**; as arrows indicated), compared to the normal conjunctiva (**B**). **G** and **H** demonstrate representative images and statistical analysis of western blotting results of IPO13 in the normal conjunctiva, primary pterygium and recurrent pterygium. The IPO13 protein level was statistically significantly increased in the pterygium and recurrent pterygium, compared to the normal conjunctiva. Data are represented as mean±SEM, n=3, ***: p<0.001 versus the conjunctiva. Scale bar: 100 μm.

We first examined the expression of IPO13 in the epithelium of the pterygium . The tissue samples from the pterygia and normal conjunctiva were analyzed with immunohistochemistry with anti-IPO13 antibody to determine the distribution of IPO13. IPO13 was highly expressed in the pterygium specimens, stained dominantly in the basal layer of epithelium, compared to the normal conjunctiva (n=10 in each group). Further, IPO13 expression was highest in the recurrent pterygium (Figure 1A-F). To confirm the increased levels of IPO13 in the pterygium, we performed western blotting with the anti-IPO13 antibody. IPO13 levels were statistically significantly increased in the primary and recurrent pterygium, compared with normal conjunctiva (n=3 in each group; Figure 1G,H). IPO13 activity was significantly increased in the epithelium of the pterygium, compared to the normal conjunctiva, suggesting an association of IPO13 with the pterygium.

### Importin 13 affected cell proliferation of cultured pterygium epithelial cells

**Figure 2:**
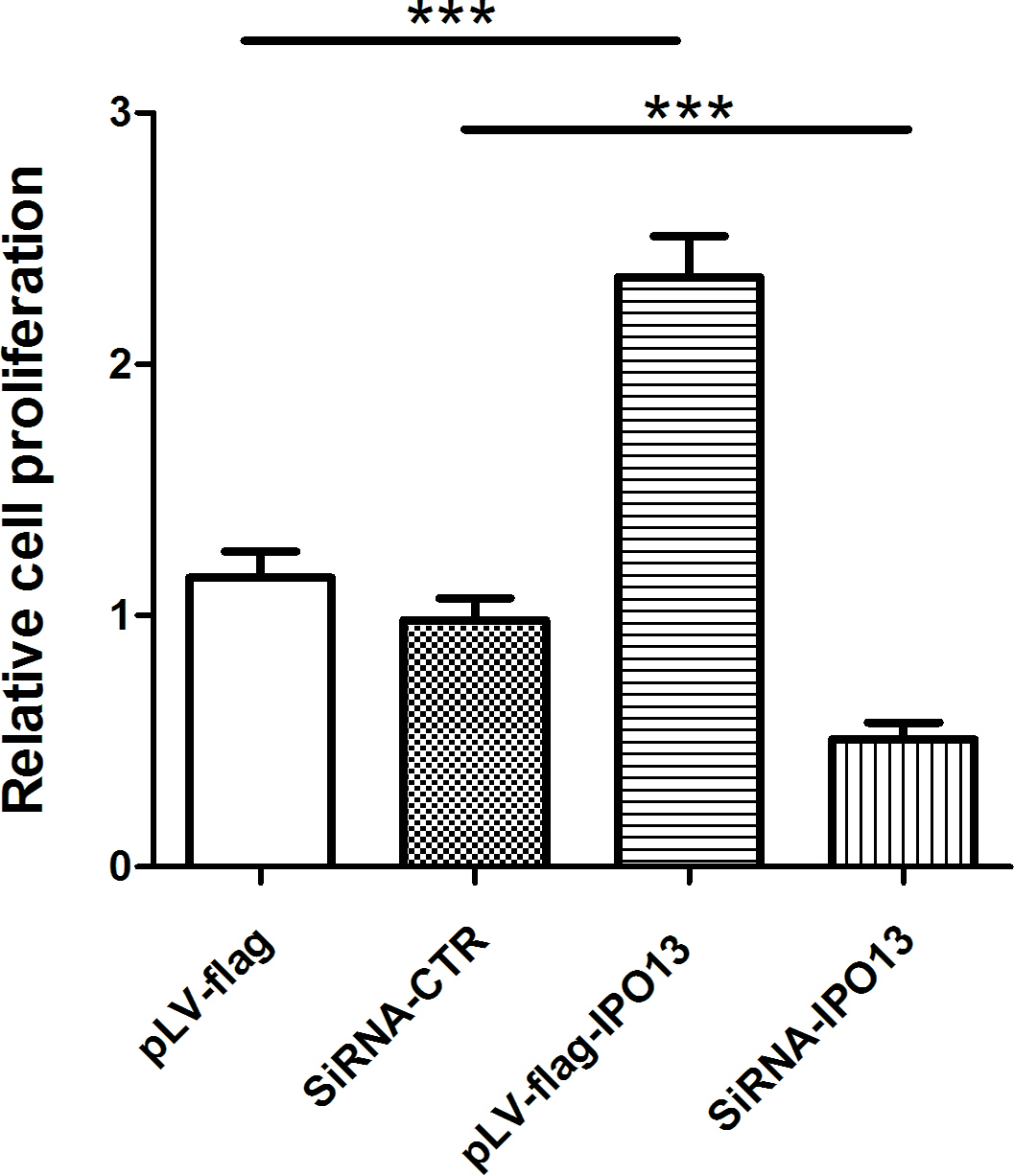
Importin 13 (IPO13) affected cell proliferation of cultured pterygium epithelial cells. Cell proliferation was assessed with 3-(4,5- dimethyl-2-thiazoyl)-2,5-di¬phenyl-2H-tetrazolium bromide (MTT) assay after transfection with pLV-flag, pLV-flag-IPO13, siRNA-CTR, and siRNA-IPO13 in the cultured pterygium epithelial cells (PECs). Overexpression of the *IPO13* gene significantly increased the proliferation of PECs compared to pLV-flag; meanwhile, knockdown of the *IPO13* gene significantly inhibited the cell proliferation of PECs compared to siRNA-CTR Data are represented as mean±SEM, n=4 in each group, *** p<0.001 versus pLV-flag or siRNA-CTR.

To elucidate the underlying mechanism of increased IPO13 activity in the epithelium of the pterygium, we first determined if IPO13 affects the cell proliferation of cultured PECs by overexpression or silencing of the *IPO13* gene, since hyperproliferation of pterygium epithelial cells is considered to partially contribute to the pathogenesis of pterygium [24-26]. As shown in Figure 2, overexpression of the *IPO13* gene statistically significantly increased the cell proliferation of cultured PECs compared to the pLV-flag group (a control of overexpression of the *IPO13* gene), suggesting that IPO13 plays a role in the cell proliferation of PECs. Meanwhile, knockdown of the *IPO13* gene statistically significantly inhibited the cell proliferation of PECs compared to the siRNA-CTR group (a control for silencing the *IPO13* gene; Figure 2).

### Colocalization of importin 13 and keratin 17 in the epithelium of the pterygium

**Figure 3:**
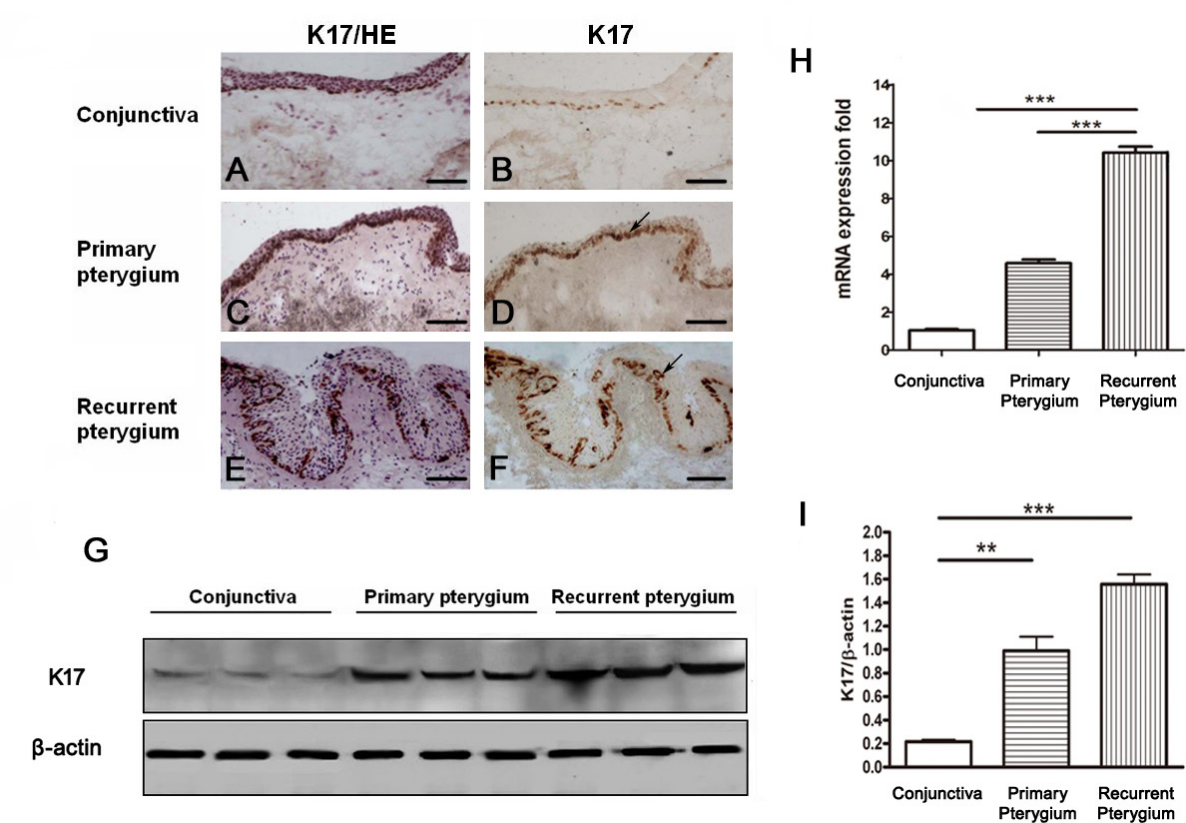
Keratin 17 (K17) expression was increased in the epithelium of the pterygium. **A-F** demonstrates representative images of immunohistochemical staining of K17 in the normal conjunctiva (**A, B**), primary pterygium (**C, D**), and recurrent pterygium (**E,F**); **A**, **C**, **E** demonstrate representative images of H&E staining indicating the nuclei in the normal conjunctiva and the pterygium. Expression of K17 in the basal layer of epithelium cells was increased (**D**, **F**; as arrows indicated), compared to the normal conjunctiva (**B**). **G** and **I** demonstrate representative images and statistical analysis of western blotting of K17 in the normal conjunctiva, primary pterygium, and recurrent pterygium. The K17 protein level was statistically significantly increased in the primary pterygium and the recurrent pterygium, compared to the normal conjunctiva. Data are represented as mean±SEM, n=3, **: p<0.01; ***:p<0.001 versus conjunctiva. **H** demonstrates quantitative real-time PCR data of K17mRNA in normal conjunctiva, primary pterygium, and recurrent pterygium. The K17 mRNA level was statistically significantly upregulated in the primary and recurrent pterygium. Data are represented as mean±SEM, n=10 in each group, *** p<0.001 versus recurrent pterygium. Scale bar: 100 μm.

To further understand the role of IPO13 in the pathogenesis of the pterygium, we next investigated the interaction of IPO13 with the cell proliferation–related factor, K17, which, as a lesional protein, is correlated with robust inflammation and epithelium proliferation [27,28]. We used immunohistochemical staining to examine K17 expression in the pterygium and the normal conjunctiva. Identical to the expression of IPO13, K17 was highly stained in the epithelium of the pterygium compared to the normal conjunctiva (n=10 in each group). Further, K17 expression was highest in the recurrent pterygium (Figure 3A-F).

We also conducted western blotting and quantitative real-time PCR to detect K17 protein levels and messenger RNA (mRNA) levels in the pterygium and normal conjunctiva. K17 protein levels of the primary pterygium and recurrent pterygium (n=3) were statistically significantly increased compared to the normal conjunctiva (n=3; Figure 3G,I). Further, the K17 protein level was even higher in the recurrent pterygium than that in the primary pterygium (Figure 3G,I). In addition, the quantitative real-time PCR data (n=10 in each group) showed that the K17 mRNA levels in the primary and recurrent pterygium were 6.4- and 12.5-fold higher than that in the normal conjunctiva (Figure 3H).

**Figure 4:**
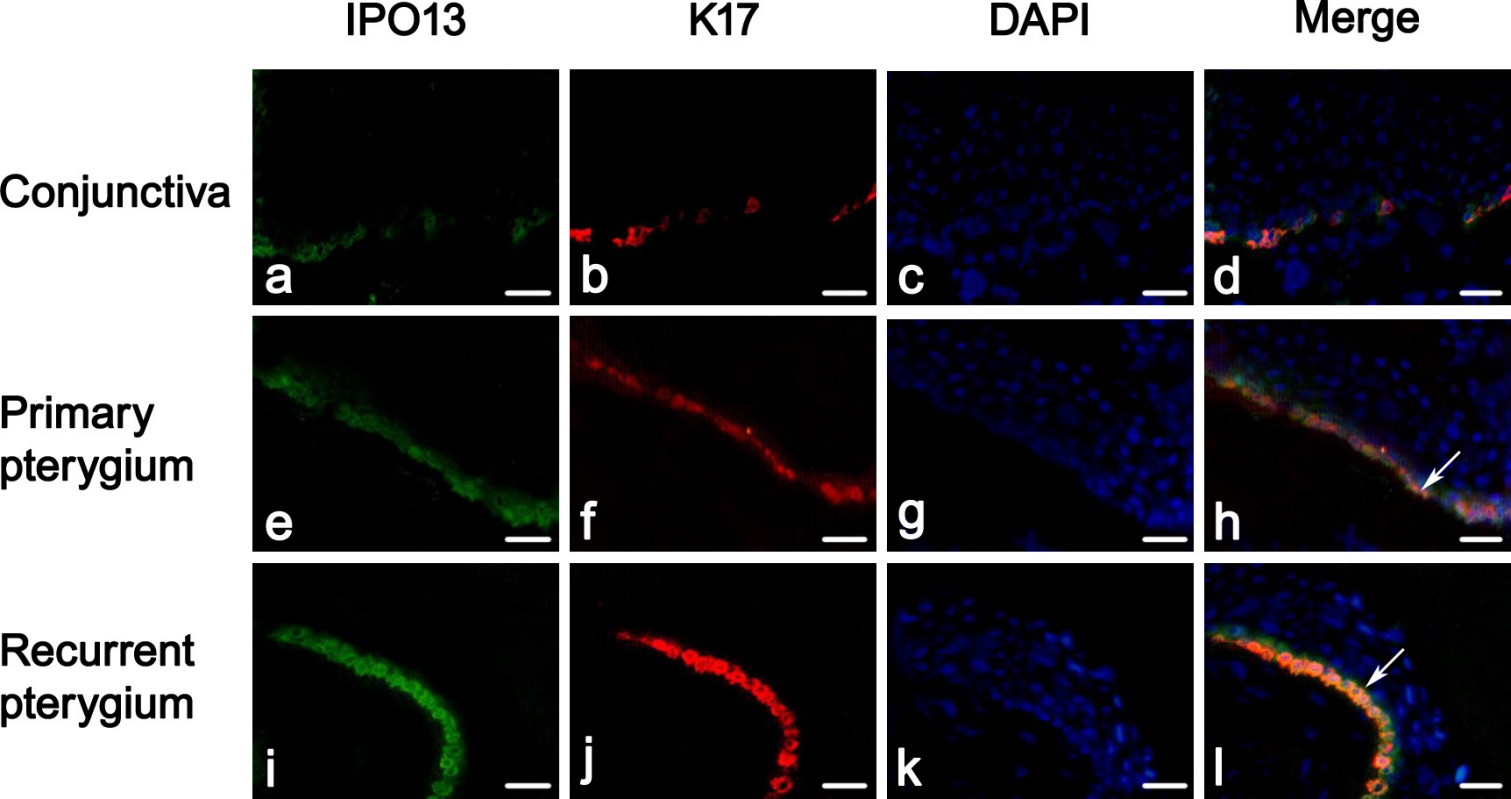
Colocalization of importin 13 and keratin 17 in the epithelium of the pterygium. Representative images of double immunofluorescence staining of importin 13 (IPO13; green) and keratin 17 (K17; red) in the normal conjunctiva, primary pterygium, and recurrent pterygium. **A**–**D** demonstrate normal conjunctiva. **E**–**H** demonstrate primary pterygium. **I**-**L** demonstrate recurrent pterygium. IPO13 was colocalized with K17 in the basal layer of the conjunctiva and the epithelium of the pterygium (as arrows indicated). Scale bar: 50 μm.

The colocalization of IPO13 with K17 was also confirmed in the basal epithelium of the pterygium with immunofluorescence double staining compared to the basal epithelium of the conjunctiva (n=8 in each group; Figure 4). The fact that IPO13 was colocalized with K17 suggested that IPO13 might be associated with K17.

### Importin 13 regulated keratin 17 expression in pterygium epithelial cells

**Figure 5:**
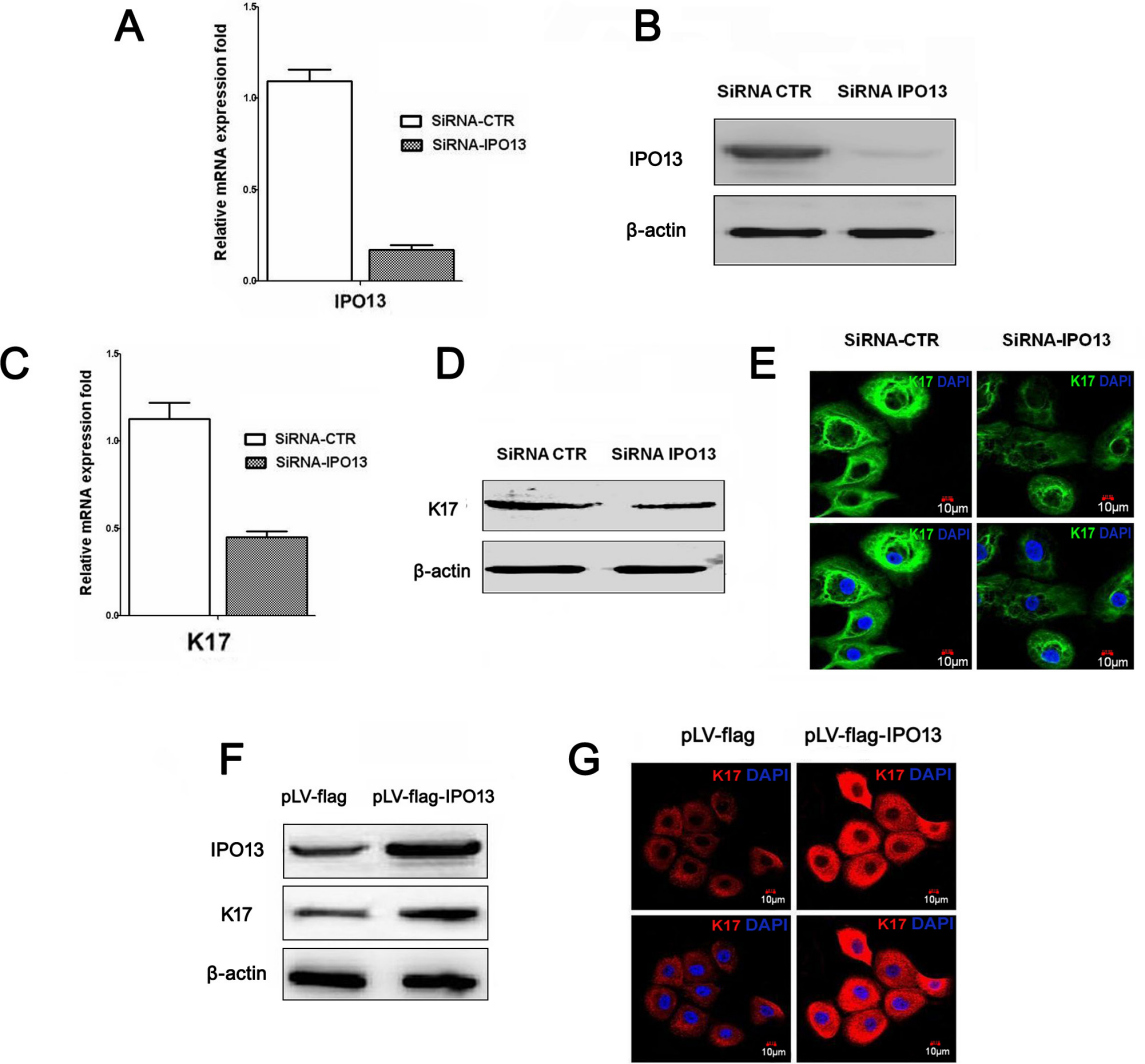
Importin 13 regulated keratin 17 in the pterygium epithelial cells. **A**–**E** demonstrate knockdown of the *IPO13* gene inhibited keratin 17 (K17) expression. **A** demonstrates quantitative real-time PCR analysis of IPO13 in the control (siRNA-CTR) and IPO13-silenced531 pterygium epithelial cells (PECs; siRNA-IPO13). **B** demonstrates Western blotting results of PECs, transfected with control and siRNA-IPO13. **C**: Quantitative real-time PCR analysis of K17 mRNA transcripts in the control and siRNA-IPO13. **D** demonstrates western blotting results of PECs transfected with control and siRNA-IPO13. **E** demonstrates representative images of immunofluorescence staining of K17 with anti-K17 antibody in the PECs transfected with control and siRNA-IPO13. DAPI (blue) counterstaining. Silencing of the *IPO13* gene significantly inhibited K17 expression. **F** and **G** demonstrate overexpression of the *IPO13* gene increased K17 expression. **F** demonstrates representative images of western blotting of PECs transfected with pLV-flag empty vector (control) and IPO13 overexpressing (pLV-flag-IPO13). **G** demonstrates representative images of immunofluorescence staining of K17 (red) with anti-K17 antibody in the PECs transfected with control and pLV-flag-IPO13. DAPI (blue) counterstaining. Overexpression of the *IPO13* gene enhanced K17 expression. Scale bar: 10 μm.

We demonstrated that IPO13 was colocalized with K17 in the epithelium of pterygium. To elucidate the interaction of IPO13 with K17, we further determined whether IPO13 regulated K17 expression in cultured PECs. The *IPO13* gene in the PECs was knocked down by transduction with a lentivirus (siRNA-IPO13) that expressed shRNA targeting IPO13. Lentivirus (siRNA-CTR)-expressing shRNA targeting the luciferase was used as control. The gene expression of IPO13 was assessed with quantitative real-time PCR. The knockdown efficiency of siRNA-IPO13 was up to 84%. The transfection of siRNA-IPO13 decreased IPO13 mRNA levels (Figure 5A). Confirming the quantitative real-time PCR assay, western blot data revealed that the siRNA-IPO13 significantly decreased IPO13 protein levels (Figure 5B).

The effect of silencing of the *IPO13* gene on K17 mRNA levels in PECs was investigated with quantitative real-time PCR after treatment with lentivirus-expressing siRNA. Quantitatively, K17 mRNA levels were reduced to 50% of control (Figure 5C). In addition, we used western blotting and immunostaining to examine the effects of silencing the *IPO13* gene on K17 protein levels in PECs. Knockdown of the *IPO13* gene induced obvious downregulation of K17 expression (Figure 5D,E).

To address whether overexpression of the *IPO13* gene increases K17 expression, we performed an overexpression experiment of the *IPO13* gene in cultured PECs by using lentiviral transduction and conducted western blotting and immunostaining. Overexpressing the *IPO13* gene upregulated the expression of K17 in PECs (Figure 5F,G). Taken together, these findings indicated that IPO13 is associated with K17 and IPO13 regulates K17 expression in PECs.

### Silencing of the importin 13 gene inhibited nuclear translocation of c-Jun

**Figure 6:**
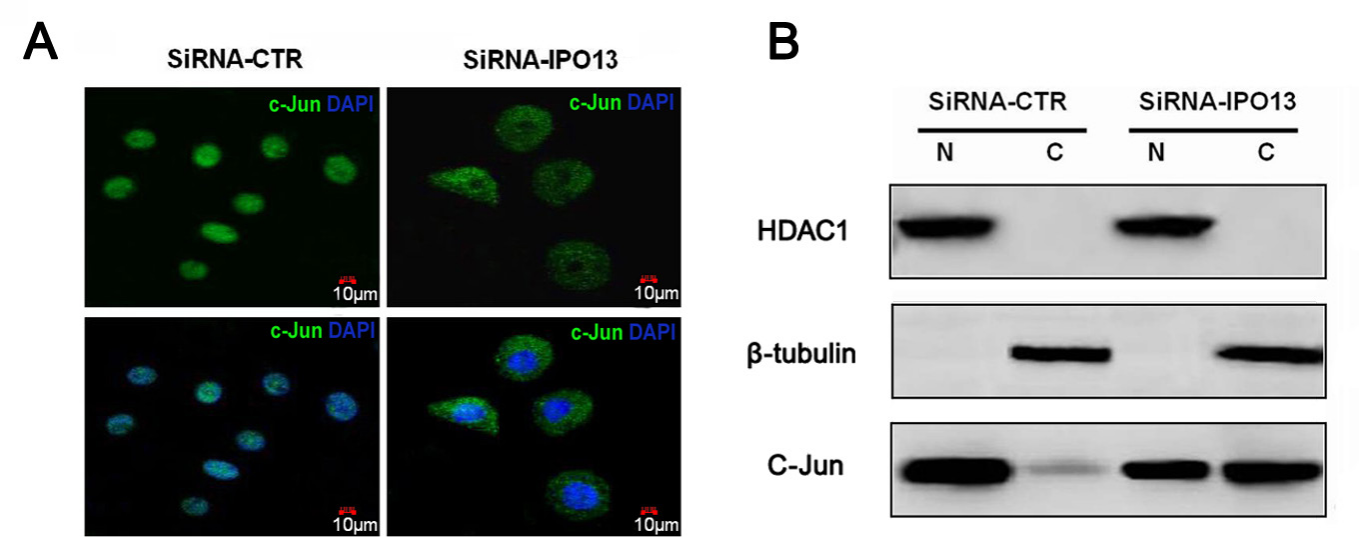
Silencing of the importin 13 gene inhibited nuclear translocation of c-Jun. **A** demonstrates representative images of immunofluorescence staining of c-Jun with anti-c-Jun antibody in the pterygium epithelial cells (PECs) after transfection with siRNA-CTR and siRNA-IPO13. DAPI (blue): counterstaining. **B** demonstrates western blotting results of c-Jun distribution in the cytoplasmic and nuclear fractions with anti-c-Jun antibody, transfected with siRNA-CTR and siRNA-IPO13. β-tubulin and HDAC1 as markers for the purity of cytoplasmic and nuclear fractions, respectively. N=nuclear fraction C=cytoplasmic fraction. Silencing of the *IPO13* gene blocked nuclear translocation of c-Jun in the PECs. Scale bar: 10 μm.

To further study the mechanism of IPO13 in the pathogenesis of the pterygium, we also investigated the interaction of IPO13 with the transcription factor c-Jun, a prototypic member of AP-1. c-Jun is also associated with cell proliferation [29]. siRNA-IPO13 efficiently prevented nuclear translocation of c-Jun, resulting in cytoplasmic retention; meanwhile, the PECs treated with siRNA-CTR (control) did not affect the nuclear import of c-Jun (Figure 6A). Moreover, the western blotting results revealed that the majority of the c-Jun protein was distributed in the nuclei of the siRNA-CTR–treated PECs, while most of c-Jun was localized in the cytoplasm of the siRNA-IPO13–transfected cells (Figure 6B). These results demonstrated that IPO13 might interact with c-Jun, and silencing of the *IPO13* gene blocked c-Jun from nuclear translocation.

## Discussion

The pathogenesis of the pterygium remains largely unknown. In the present study, we for the first time investigated the role of IPO13, a member of the importin-β family of nuclear import proteins, in the pathogenesis of the pterygium and the interaction of IPO13 and other cell proliferation–related factors, K17 and c-Jun. In contrast to the normal conjunctiva, IPO13 activity was significantly increased in the epithelium of the pterygium. IPO13 also regulated the cell proliferation of cultured PECs. However, IPO13 was colocalized with K17 in the epithelium of the pterygium. Knockdown or overexpression of the *IPO13* gene affected K17 expression in PECs. In addition, knockdown of the *IPO13* gene blocked the nuclear translocation of c-Jun. Collectively, these findings indicate that IPO13 plays a role in the pathogenesis of pterygia and the underlying mechanism of IPO13 in the pterygium is via regulating the expression of K17 and nuclear entry of c-Jun. This study provides new insights into the pathogenesis of the pterygium and may contribute to the development of new therapeutic agents for pterygia.

As cell hyperproliferation partially contributes to the pathogenesis of the pterygium [24-26], we examined and focused on the effects of IPO13 on cell proliferation of the pterygium using cultured PECs. We investigated the effects of overexpression and knockdown of the *IPO13* gene on the cell proliferation of PECs. We showed that overexpression of the *IPO13* gene can promote cell growth, whereas knockdown of the *IPO13* gene significantly inhibited cell proliferation. This is the first evidence that silencing of the *IPO13* gene has an inhibitory effect on the cell proliferation of PECs, thus providing a new potential direction for antipterygium treatment.

Pterygium is proliferative and highly vascularized tissue. It has been believed or hypothesized that the pathogenesis of pterygium is caused by DNA damage, oxidative stress, limbal stem cell deficiency, etc. [30-32]. Recently, many researchers have focused on the role of cell proliferation; it is believed that cell hyperproliferation of the epithelium leads to a pterygium. Kase et al. applied a set of reliable cycle-related markers (P27, Ki67, cyclin D1) to investigate pterygium and conjunctival tissues, and showed that these markers are present in the epithelium of the pterygium but are absent in the pterygium stroma [24]. Kase et al. proposed that the development of a pterygium is associated with cell proliferation [24]. Recently, Liang et al. analyzed the expression pattern of cell proliferation molecules in pterygium specimens from Chinese patients and showed direct agreement with previous reports [24-26]. Therefore, previous research has suggested that cell proliferation of the epithelium is associated with the pathogenesis of the pterygium, and our present investigation supported this hypothesis. Although we showed increased IPO13 and K17 expression, which is related to cell proliferation, in the pterygium specimens, further investigations, including use of more specific cell proliferation factors, markers, or other approaches, are needed to better elucidate the role of cell proliferation in the pathogenesis of the pterygium.

The import of many proteins to the nucleus is mediated by members of the family of evolutionarily conserved transport factors, the importin-β (also called karyopherin ß) family. IPO13 is a member of the importin-β family of nuclear import proteins. Previous studies revealed that most cargo substrates for IPO13 are transcriptional factors. Ploski et al. reported that IPO13 mediated the nuclear import of proteins (Pax6, Pax3, and Crx) [4]. We previously reported that IPO13 regulates nuclear import of the glucocorticoid receptor in airway epithelial cells [6]. We recently showed that IPO13 can serve as a potential marker for corneal epithelial progenitor cells and IPO13 is associated with corneal cell proliferation [7]. In the present study, we continuously investigated the role of IPO13, by focusing on the epithelium of the pterygium and the interaction of IPO13 with other cell proliferation–related factors, K17 and c-Jun. Recently, researchers preferentially observed K17 as a pathogenic effector involved in disease occurrence or deterioration due to K17’s proliferative feature [27,28]. Our evidence suggested that IPO13 might play a role in the cell proliferation of the pterygium via regulating K17 expression. As a nucleocytoplasmic transport receptor, IPO13 may not modulate K17 expression directly. We hypothesized that IPO13 may modulate the nuclear translocation of cargo substrates including K17 regulators, for example, c-Jun. c-Jun, a prototypic member of AP-1, controls the expression of cytokines and chemokines, and c-Jun is causally involved in inflammatory skin diseases such as psoriasis [17,18]. Waldmann et al. recently demonstrated that c-Jun can interact with IPO13 in in vitro assay [33]. In our lentivirus-mediated silencing of *IPO13* gene experiments, downregulation of IPO13 inhibited nuclear translocation of c-Jun, supporting IPO13 regulation of c-Jun activity. Collectively, these findings suggest that IPO13 potentiates the regulation of K17 probably through promoting nuclear translocation of c-Jun. However, IPO13 might be partially responsible for K17 expression, and other mechanisms for modulating K17 remain to be investigated.

We revealed that IPO13 and K17 expression was higher in the recurrent pterygium than that in the primary pterygium in immunostaining and western blot analysis. This difference may be due to by the higher cell proliferation pattern in the recurrent the pterygium than the primary pterygium, but must be further elucidated.

In the present study, we demonstrated that IPO13 may play a role in the pathogenesis of pterygium, and the underlying mechanism may be through modulation of K17 and c-Jun. The evidence presented provides a hint that suppressing IPO13 may have a potential therapeutic effect on the development of the pterygium.
